# Development and Validation of Machine Learning Models for Predicting Low Birth Weight in Singleton Pregnancies in Vietnam

**DOI:** 10.7759/cureus.106995

**Published:** 2026-04-13

**Authors:** Rang N Nguyen, Thuyen K Truong, Tri H Ngo

**Affiliations:** 1 Pediatrics, Can Tho University of Medicine and Pharmacy, Cần Thơ, VNM; 2 Obstetrics and Gynecology, An Giang Hospital of Obstetrics, Gynecology and Pediatrics, An Giang, VNM; 3 Pediatrics, An Giang Hospital of Obstetrics, Gynecology and Pediatrics, An Giang, VNM

**Keywords:** cardiotocography, low birth weight, machine learning, preterm, random forest

## Abstract

Objective: This study compared seven machine learning (ML) algorithms to identify the most effective model for predicting low birth weight (LBW) in singleton pregnancies. The primary goal was to develop a high-accuracy screening tool to support clinical decision-making and early intervention.

Methods: A prospective cross-sectional study was conducted among women delivering at the Women and Children Hospital of An Giang, Vietnam. Feature selection was performed using the Boruta algorithm, and data imbalance was addressed with the Synthetic Minority Over-sampling Technique (SMOTE). Seven ML algorithms - logistic regression (LR), random forest (RF), support vector machine, k-nearest neighbor, naïve Bayes, artificial neural network, and XGBoost (Seattle, WA: University of Washington) - were trained using five-fold cross-validation. Model performance was assessed using area under the curve (AUC), accuracy, sensitivity, and specificity, while SHapley Additive exPlanations (SHAP) analysis was applied to interpret feature importance and explain the final model.

Results: Among 1,838 women with singleton pregnancies (1,678 non-LBW and 160 LBW), the prevalence of LBW was 8.7% (95% CI: 7.5-10.0%). Of the seven ML models evaluated, the RF model achieved the highest overall performance, with an AUC of 0.778, an accuracy of 0.871, and a specificity of 0.909. LR demonstrated the highest sensitivity (0.581). SHAP analysis of the RF model identified preterm birth as the most important predictor of LBW, followed by primiparity, absence of gestational diabetes, abnormal cardiotocography (CTG) findings, pre-eclampsia, and a prior history of LBW.

Conclusion: ML-based prediction of LBW enables early identification of high-risk pregnancies and enables timely preventive strategies. In this study, the RF model demonstrated the best predictive performance, with key predictors including preterm birth, primiparity (first-born status), absence of gestational diabetes, abnormal CTG finding, pre-eclampsia, and prior history of LBW. Early identification combined with appropriate perinatal and neonatal care may reduce infant mortality and severe morbidity.

## Introduction

Low birth weight (LBW) is defined by the World Health Organization (WHO) as a birth weight below 2.5 kg and remains a major global public health concern and an important indicator of maternal and neonatal health [1]. Each year, approximately 20 million infants, accounting for 15-20% of all live births worldwide, are born with LBW, with prevalence more than twice as high in developing countries as in developed nations [2]. LBW has profound consequences, leading to higher morbidity and mortality risks throughout an infant’s life compared to infants of normal weight. In addition to increased neonatal mortality, LBW is associated with impaired growth, delayed cognitive development, and an elevated risk of chronic diseases later in life, including diabetes, obesity, and cardiovascular disorders [3].

In developed nations, preterm birth is the primary cause of LBW, while in low- and middle-income countries, LBW is a complex outcome influenced by a confluence of sociodemographic, maternal, and medical factors. Sociodemographic determinants significantly contribute to risk, including living in a rural area, experiencing low income, having limited educational attainment, and engaging in heavy manual labor. Maternal factors further contribute to vulnerability, particularly extremes of maternal age (too young or too advanced), short intervals between pregnancies, low maternal height and weight, inadequate nutritional intake during gestation, gestational anemia, and insufficient antenatal care visits. Furthermore, specific maternal illnesses, particularly gestational hypertension and pre-eclampsia, are well-established conditions that further elevate the probability of delivering an infant with LBW [4-8].

Over the past few years, machine learning (ML), a central domain within artificial intelligence, has emerged as a powerful tool for medical diagnosis and outcome prediction. In contrast to conventional statistical approaches, machine learning algorithms can handle large and complex datasets without requiring assumptions about data distribution, making them well-suited for identifying intricate, non-linear relationships among numerous risk factors. Previous studies have successfully employed various ML classifiers, such as logistic regression (LR), random forest (RF), and Extreme Gradient Boosting (XGBoost; Seattle, WA: University of Washington), to predict LBW across diverse populations, demonstrating high accuracy and the ability to pinpoint the most influential predictors. These models consistently identify preterm delivery as the most influential predictor of LBW, followed by crucial maternal factors, including biometrics such as weight, height, and BMI; maternal morbidities during pregnancy like hypertension, pre-eclampsia, and gestational diabetes; and obstetric history (previous preterm birth, LBW, and abortion). Other significant contributors included the adequacy of antenatal care coverage, infant-related factors such as sex and birth order, and various socioeconomic and demographic variables like place of birth, family income, education level, and occupation involving heavy labor [4,9-12].

In Vietnam, the prevalence of LBW varies between 7.9% and 12.5%. Several protective factors have been found to be negatively associated with LBW, including higher maternal body mass index, maintaining good nutritional status during pregnancy, and taking maternity leave before delivery [13]. Despite the growing application of ML in obstetrics, there is currently a lack of ML-based prediction models for LBW in the Vietnamese population. Traditional statistical methods may be limited in capturing complex, non-linear relationships among multiple maternal and clinical factors, whereas ML approaches can effectively handle high-dimensional data and uncover intricate interactions between predictors. Therefore, the primary objective of this study was to identify the most effective ML algorithm for the early prediction of LBW to support clinical decision-making. By comparing seven different models, we aimed to develop a high-accuracy screening tool for singleton pregnancies, with the primary outcome defined as a birth weight of less than 2,500 g.

## Materials and methods

Study design, setting, and participants

A prospective cross-sectional study was conducted among pregnant women and their newborns admitted for delivery at the Women and Children Hospital of An Giang, Vietnam, from January 2025 to July 2025. We recruited all eligible subjects until the required sample size was reached, excluding multiple births (twins or higher-order multiples) and infants with congenital malformations.

Inclusion and exclusion criteria

This study included pregnant women with singleton pregnancies who provided informed consent and underwent cardiotocography (CTG) monitoring prior to delivery. We excluded multiple gestations (twins or higher-order multiples) and infants with congenital malformations to ensure the accuracy of the predictive model.

Sample size

Based on seven predictor variables and an 8.2% LBW prevalence, a minimum sample size of 1,708 was required to maintain a conservative ratio of 20 events per feature [14]. We employed a convenience sampling technique, where all eligible pregnant women admitted for delivery were recruited consecutively until the predetermined sample size was reached.

Data collection

We collected data via direct interviews with pregnant women in the delivery room and through medical record reviews covering the period from admission until discharge. Comprehensive data were gathered across demographic, obstetric, and neonatal domains. Demographic variables included age, occupation, education, residence, and BMI-related metrics (height and weight). Pregnancy-specific data covered obstetric history (previous births, LBW history, miscarriages, and cesarean sections), prenatal visit frequency, and secondhand smoke exposure, alongside comorbidities like pre-eclampsia and gestational diabetes. Finally, neonatal characteristics, specifically sex, gestational age, and birth weight, were recorded. All patients received cardiotocography prior to delivery.

Outcome variable

Low birth weight (LBW) was defined as a birth weight of below 2,500 g according to the World Health Organization (WHO) standards. Infants were categorized using a binary outcome variable, with one indicating LBW and 0 indicating normal birth weight (NBW). To ensure data integrity, weights were measured immediately postpartum by trained clinical staff using calibrated scales.

Predictor variables

To select the predictor variables for our model, we initially identified 18 factors based on a comprehensive review of existing literature and established clinical studies [15-17]. These variables spanned the following four primary domains: demographic characteristics, maternal biometrics, obstetric history, and current clinical findings, including pre-eclampsia and cardiotocography (CTG) results. To ensure consistency across the machine learning pipeline, all predictors were dichotomized and numerically encoded. Clinical risk factors were assigned a value of '1' for presence and '0' for absence, establishing the latter as the reference category. The following 18 selected variables are: maternal age (0: ≤35, 1: >35 years), occupation (0: housewife, 1: employed), education level (1: primary, 0: secondary), place of residence (0: urban, 1: rural), height (0: ≥150 cm, 1: <150 cm), weight (0: ≥50 kg, 1: <50 kg), parity (1: primiparity, 0: multiparity: ≥2), history of low birth weight (1: yes, 0: no), history of C-section (1: yes, 0: no), antenatal care visits (1: <4 visits, 0: ≥4 visits), exposure to secondhand smoke (1: yes, 0: no), pre-eclampsia (1: yes, 0: no), gestational diabetes (1: yes, 0: no), gestational weight gain (1: <10 kg, 0: ≥10 kg), preterm birth (1: yes, 0: no), and CTG (0: normal, 1: abnormal).

Cardiotocography (CTG) was performed for all participants prior to delivery as part of routine obstetric care. CTG tracings were interpreted in line with the guidelines issued by the International Federation of Gynecology and Obstetrics (FIGO). CTG findings were classified as normal or abnormal based on fetal heart rate baseline, variability, accelerations, decelerations, and uterine contraction patterns. An abnormal CTG was defined as any tracing demonstrating non-reassuring features suggestive of potential fetal compromise. CTG interpretation was conducted by trained obstetric staff, and the final classification was recorded in the medical records and used for analysis [18].

Data preprocessing

Continuous variables were screened for outliers using boxplots to visualize and evaluate distribution anomalies. Missing values in the dataset were addressed using the mice package in R. This imputation step was essential to ensure data completeness and integrity before modeling. Before performing feature selection, the dataset was randomly split into training and testing subsets in a 70:30 proportion.

Feature selection

The Boruta algorithm was applied to identify and retain the most relevant variables. To prevent data leakage and ensure an unbiased evaluation of the final model, this process was strictly performed only on the training set [19].

Dataset balancing

A significant class imbalance was present in the dataset, characterized by a low number of LBW cases. Therefore, the Synthetic Minority Over-sampling Technique (SMOTE) was implemented on the training dataset. This technique allows machine learning models to effectively capture the characteristics of the rare outcome, improving sensitivity while maintaining generalization and preventing overfitting [20].

Model evaluation

Seven ML classification algorithms were trained on the training dataset, including logistic regression (LR), random forest (RF), k-nearest neighbor (KNN), naïve Bayes (NB), support vector machine (SVM), artificial neural network (ANN), and Extreme Gradient Boosting (XGBoost). The models were trained on the training set and evaluated on the test set. To maximize the predictive performance of the seven ML algorithms, we performed extensive hyperparameter tuning using a grid search approach provided by the caret package in R. For each model, a predefined search space of key hyperparameters was established. To ensure the stability and generalizability of the tuned parameters, we employed five-fold cross-validation during the optimization process. The final model for each algorithm was selected based on the configuration that yielded the highest area under the receiver operating characteristic curve (AUROC) on the validation sets. Model performance was thoroughly assessed using a range of classification metrics, including accuracy, sensitivity (recall), specificity, and both positive and negative predictive values (PPV and NPV).

Model interpretations

The SHapley Additive exPlanations (SHAP) method was applied to enhance model interpretability by assigning consistent, locally accurate contribution scores (SHAP values) to each feature. Higher SHAP values correspond to an increased probability of LBW. The overall impact of each predictor on LBW risk was derived by aggregating its SHAP contributions across all observations [21].

Statistical analyses

Categorical variables were described using frequencies and percentages, and group comparisons were conducted using the chi-square test or Fisher’s exact test, as appropriate. A two-tailed p-value below 0.05 was considered indicative of statistical significance. All statistical analyses and ML implementations were performed using R software version 4.4.1 (Vienna, Austria: R Foundation for Statistical Computing) and relevant packages, such as caret, Boruta, smotefamily, e1071, kernlab, randomForest, nnet, and xgboost.

## Results

Baseline characteristics

**Figure 1:**
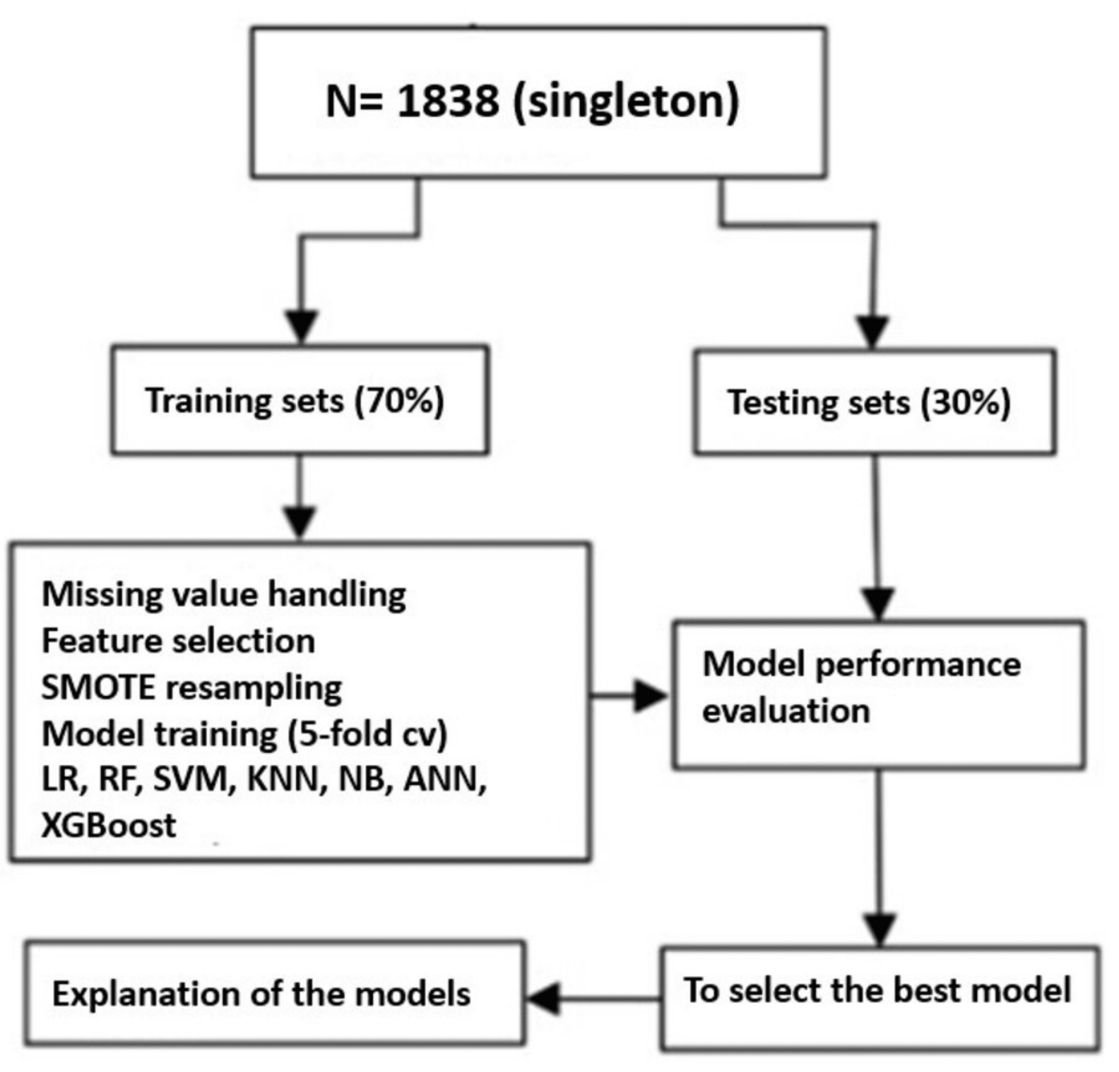
Flow chart of the study design. LR: logistic regression; RF: random forest; SVM: support vector machine; KNN: k-nearest neighbor; NB: naïve Bayes; ANN: artificial neural network; XGBoost: Extreme Gradient Boosting

A total of 1,859 cases were initially recorded. After excluding 21 cases involving congenital malformations or multiple pregnancies, 1,838 singleton pregnancies were included in the final analysis. Among these, 160 infants were classified as low birth weight (LBW), representing a prevalence of 8.7%. The flowchart is shown in Figure 1.

Demographic and clinical characteristics of study participants

Mothers in the LBW group were significantly more likely to have a height ≤150 cm and a weight ≤50 kg compared with the normal birth weight (NBW) group (both p<0.01). Lower maternal education at the primary level was also more frequent among LBW cases (p=0.042). No significant differences were observed between groups regarding maternal age >35 years, rural residence, occupation, or passive smoking. Regarding obstetric and clinical factors, primiparity, previous history of LBW, pre-eclampsia, fewer than four antenatal care visits, and preterm delivery were all significantly more common in the LBW group (all p<0.05). Abnormal cardiotocography (CTG) findings were also significantly higher among LBW neonates (p<0.001). In contrast, gestational diabetes and adequate maternal weight gain during pregnancy were more frequent in the NBW group (both p<0.05). There were no significant differences between groups in previous abortion, previous cesarean section, or infant sex (Table 1).

**Table 1:** Demographic and clinical characteristics of study participants. *Chi-square test. LBW: low birth weight; NBW: normal birth weight; CTG: cardiotocography

Characteristics	NBW (n=1678)	LBW (n=160)	Total (n=1838)	p-Value
Maternal characteristics				
Maternal age >35 years, n (%)	259 (15.4)	27 (16.9)	286 (15.6)	0.714
Maternal height ≤150 cm, n (%)	317 (18.9)	46 (28.7)	363 (19.7)	0.003*
Maternal weight ≤50 kg, n (%)	86 (5.1)	22 (13.8)	108 (5.9)	<0.001*
Rural residence, n (%)	1435 (85.5)	136 (85.0)	1571 (85.5)	0.915
Occupation (housewife), n (%)	686 (40.9)	72 (45.0)	758 (41.2)	0.354
Education (primary level), n (%)	245 (14.6)	33 (20.6)	278 (15.1)	0.042*
Passive smoking: yes, n (%)	275 (16.4)	31 (19.4)	306 (16.6)	0.333
Obstetric and clinical factors				
Primiparous (first-born), n (%)	681 (40.6)	78 (48.8)	759 (41.3)	0.045*
Previous LBW history: yes, n (%)	46 (2.7)	18 (11.3)	64 (3.5)	<0.001*
Previous abortion: yes, n (%)	354 (21.1)	31 (19.4)	385 (20.9)	0.609
Previous cesarean: yes, n (%)	319 (19.0)	24 (15.0)	343 (18.7)	0.213
Pre-eclampsia: yes, n (%)	39 (2.3)	14 (8.8)	53 (2.9)	<0.001*
Gestational diabetes: yes, n (%)	96 (5.7)	1 (0.6)	97 (5.3)	<0.001*
Antenatal care visits <4, n (%)	89 (5.3)	18 (11.3)	107 (5.8)	0.002*
Maternal weight gain: yes, n (%)	519 (30.9)	35 (21.9)	554 (30.1)	0.017*
Child’s sex (female), n (%)	784 (46.7)	75 (46.9)	859 (46.7)	0.970
Preterm delivery: yes, n (%)	162 (9.7)	82 (51.2)	244 (13.3)	<0.001*
Abnormal CTG findings, n (%)	93 (5.5)	25 (15.6)	118 (6.4)	<0.001*

Feature selection

**Figure 2:**
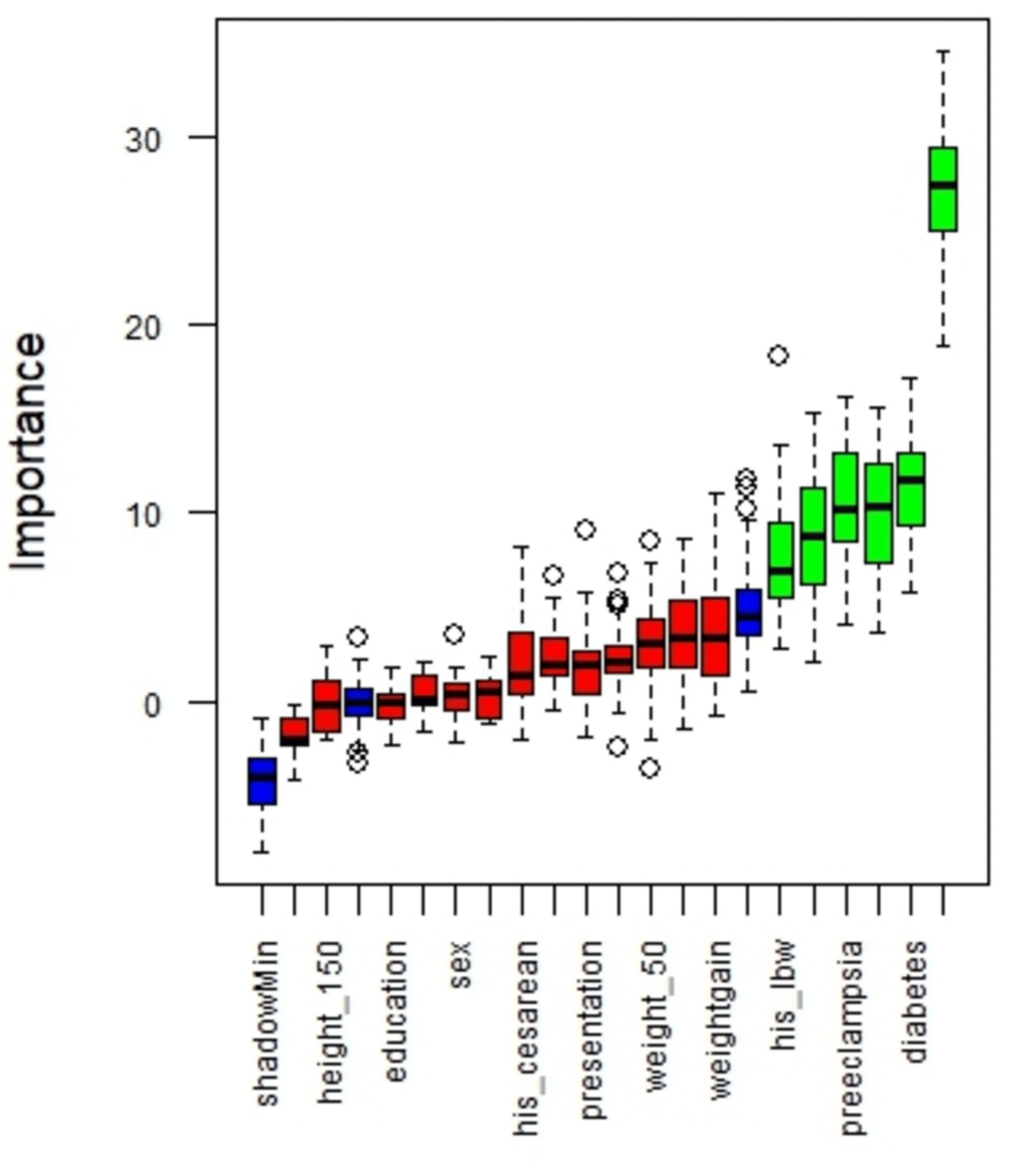
Feature selection based on the Boruta algorithm. The importance of each variable is represented by its Z-score on the vertical axis. The boxplots depict the variation of these scores across iterations of the algorithm. Variables highlighted in green indicate the six most influential features, whereas those shown in red represent features with no significant contribution to the model. his_lbw: history of low birth weight

The results of feature selection using the Boruta algorithm are presented in Figure 2. In order of Z-score values, the six variables most closely associated with LBW were pre-eclampsia, parity, gestational diabetes, previous LBW, preterm birth, and abnormal CTG.

Performance comparisons of ML-based models

**Figure 3:**
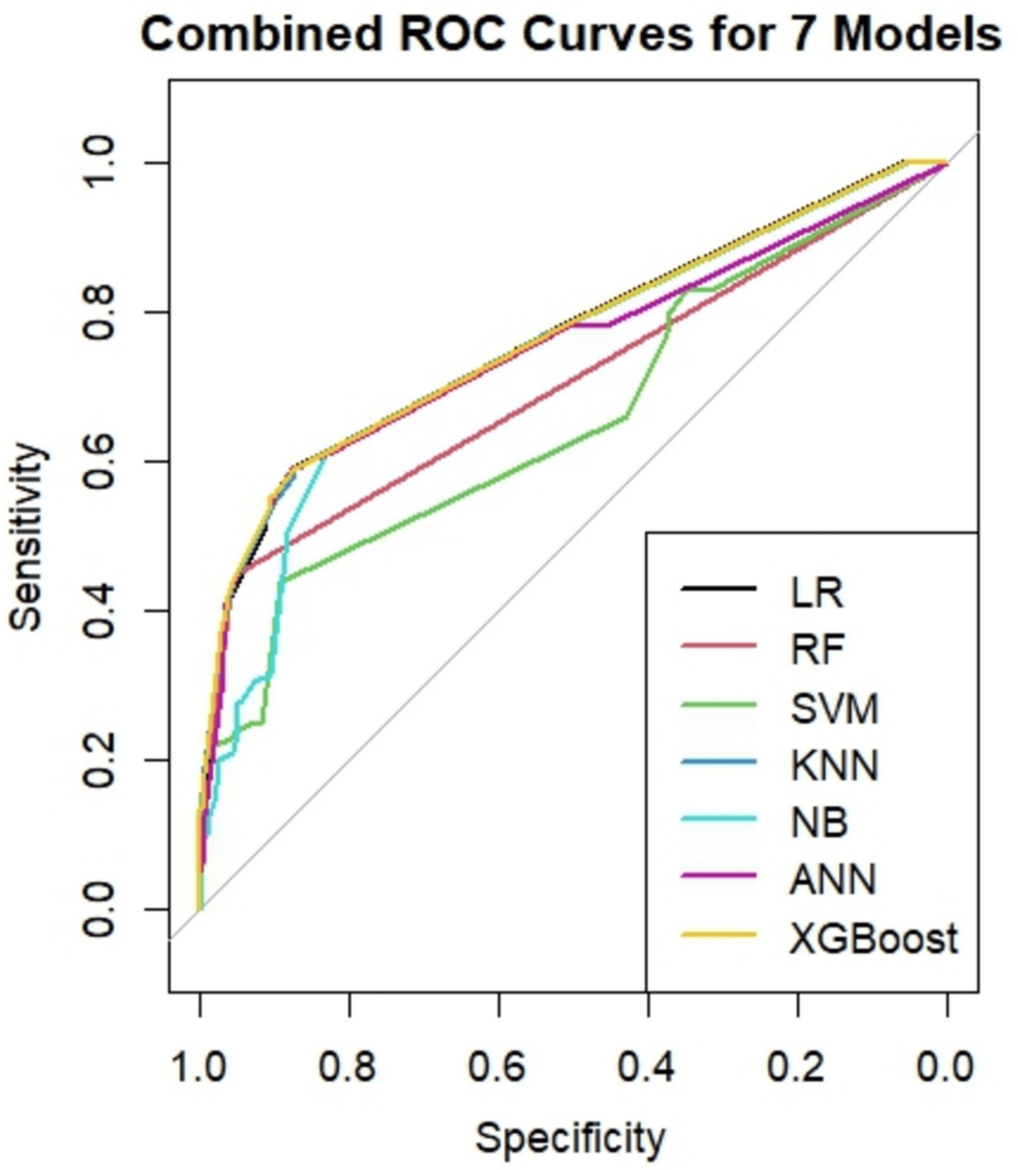
Performance by ROC curve of seven ML models for LBW prediction in the test set. LR: logistic regression; RF: random forest; SVM: support vector machine; KNN: k-nearest neighbor; NB: naïve Bayes; ANN: artificial neural network; ROC: receiver operating characteristic; ML: machine learning; LBW: low birth weight

We generated seven ML models to predict the development of LBW in singleton babies. The performance of the ML models is summarized in Table 2. Among the seven models, the random forest (RF) model achieved the highest predictive performance for LBW (AUC=0.778), followed by LR (AUC=0.775), ANN (AUC=0.745), SVM (AUC=0.725), XGBoost (AUC=0.716), KNN (AUC=0.715), and NB (AUC=0.715). Besides the area under the receiver operating characteristic curve (AUROC), additional performance metrics were also evaluated. RF achieved the highest overall accuracy (0.871), while LR provided the best sensitivity (0.581) (Table 2). The ROC curves visually illustrated similar performance between the models, with all curves approaching the top-left corner, indicating high discriminatory capacity (Figure 3).

**Table 2:** Performance metrics of machine learning models for predicting LBW in the test set. LR: logistic regression; SVM: support vector machine; KNN: k-nearest neighbor; ANN: artificial neural network; AUC: area under the ROC curve; PPV: positive predictive value; NPV: negative predictive value

Variables	AUC (CI 95%)	Accuracy (CI 95%)	Sensitivity	Specificity	PPV	NPV
LR	0.775 (0.703-0.847)	0.824 (0.789-0.854)	0.581	0.850	0.301	0.948
Random forest	0.778 (0.708-0.849)	0.871 (0.840-0.899)	0.527	0.909	0.391	0.945
SVM	0.725 (0.640-0.810)	0.856 (0.824, 0.884)	0.527	0.893	0.353	0.944
KNN	0.715 (0.628-0.802)	0.865 (0.834, 0.893)	0.527	0.903	0.376	0.945
Naïve Bayes	0.715 (0.628-0.802)	0.865 (0.834-0.893)	0.527	0.903	0.376	0.945
ANN	0.745 (0.6634-0.826)	0.854 (0.822, 0.883)	0.563	0.887	0.356	0.948
XGBoost	0.716 (0.629-0.804)	0.869 (0.838, 0.896)	0.527	0.907	0.386	0.945

Model explanation

**Figure 4:**
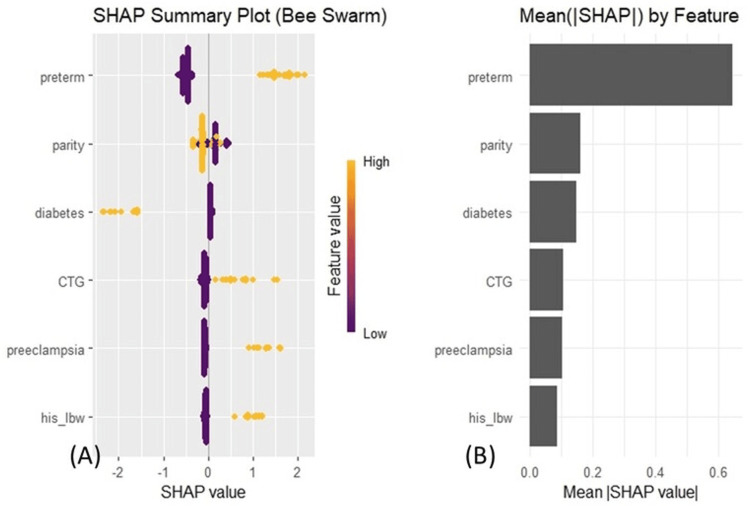
Feature importance and contribution to the predictive model. (A) SHAP summary plot (bee swarm): each point represents an individual patient, with the color indicating the feature value (yellow for high, purple for low) and its position on the x-axis representing its impact on the model's prediction. (B) Mean absolute SHAP values ranking features by their overall contribution, with preterm birth as the most important predictor. CTG: cardiotocography; his_lbw: history of low birth weight; SHAP: SHapley Additive exPlanations

SHAP was applied to interpret the final model by quantifying each variable’s contribution to the predictions. This approach provides both global (feature-level importance) and local (individual-level) explanations. As shown in Figures 4A, 4B, the most influential predictors, in descending order, were preterm birth, parity, gestational diabetes, abnormal CTG, pre-eclampsia, and prior history of LBW.

## Discussion

This study identified an LBW prevalence of 8.7% (95% CI: 7.5-10.0%), consistent with Southeast Asian data but markedly lower than the 27.1-44.5% range reported in African and South Asian countries [2]. To evaluate different ML algorithms in predicting LBW, we found that the random forest (RF) classification model demonstrated superior diagnostic performance, with an AUROC of 0.778, an accuracy of 0.871, and a specificity of 0.909, while the logistic regression (LR) model yielded the highest sensitivity at 0.581.

The performance of our RF model demonstrated comparability with several existing ML studies focused on predicting LBW across Iran, Bangladesh, and other low- and middle-income countries. However, findings regarding the optimal model in Iran were varied as follows: in a study of 741 mother-newborn pairs, Arayeshgari et al. reported that the LR model showed the best predictive capability, achieving an accuracy of 88% [9]. Conversely, Ahmadi et al. found that RF outperformed LR, achieving a superior accuracy of 95% and an AUROC of 93% [22]. Adding to this diversity of results, Ranjbar et al. analyzed 1,280 cases and concluded that Extreme Gradient Boosting (XGBoost) was the most effective predictor for LBW, though with more moderate metrics - accuracy of 0.79, precision of 0.87, recall of 0.69, and an F1 score of 0.7 [10].

In Bangladesh, a study presented conflicting findings: Pollob et al. identified the LR model as the best performer in predicting LBW, reporting an accuracy of 87.6% and an AUROC of 59.0% [23]. Conversely, a larger study of 3,192 women by Sultana et al. identified XGBoost as the top-performing model. It achieved superior results across all key metrics, specifically an accuracy of 0.80, an AUC of 0.76, and a recall of 0.80 [24].

Using a large dataset comprising 145,206 women with singleton live births, Patterson et al. conducted a multi-country evaluation of five ML models across the Democratic Republic of Congo, Kenya, Zambia, Guatemala, Pakistan, India, and Bangladesh. LR demonstrated the best overall performance, achieving an AUC of 72%, an accuracy of 61%, and a sensitivity of 72% [8].

In the present study, preterm birth emerged as the most influential predictor of LBW in the RF model, followed by primiparity (first-born status), absence of gestational diabetes, abnormal CTG finding, pre-eclampsia, and prior history of LBW. Our findings align with research from Western and South Asia, where gestational age consistently emerges as the primary predictor of LBW. In the UAE, Khan et al. utilized LR to identify gestational age, diabetes, and hypertension as key factors [11]. Similarly, Ranjbar et al. found it to be a critical predictor alongside prior LBW history in Iran [10], while Sultana et al. employed XGBoost in Bangladesh to highlight the importance of gestational age and geographic location [24].

In our study, socioeconomic factors did not significantly influence LBW, possibly reflecting substantial improvements in economic conditions and access to maternal healthcare in Vietnam since the post-Doi Moi period [25]. This finding contrasts with LBW prediction models from other developing countries, where socioeconomic variables such as place of residence, maternal education, occupation, and income level have been consistently identified as important predictors [4,8,23,26].

The clinical value of this model lies in its potential to be integrated into hospital information systems or mobile health platforms as a real-time decision-support tool. By using SHapley Additive exPlanations (SHAP) to enhance model interpretability, clinicians can go beyond “black-box” predictions and gain insight into the specific risk factors that contribute to an individual patient’s risk score. In practice, this level of transparency enables healthcare providers to deliver more targeted clinical interventions and provide more informed patient counseling. From a public health perspective, an interpretable tool like this can support resource-limited settings by helping prioritize intensive neonatal care and specialist consultations for high-risk cases, ultimately improving maternal and fetal outcomes through data-driven precision medicine.

Furthermore, the clinical utility of our model is underscored by its integration as a robust decision-support tool within the obstetric workflow. Given the relatively low prevalence of low birth weight (LBW), the model’s high specificity and negative predictive value (NPV) offer substantial value for risk de-escalation, allowing clinicians to confidently identify low-risk pregnancies and avoid unnecessary interventions. Conversely, by prioritizing high sensitivity at optimized probability thresholds, the model functions as an early-warning system that alerts providers to at-risk neonates who require intensified monitoring. This approach transforms complex ML outputs into actionable clinical insights, optimizing resource allocation and enhancing the safety of bedside decision-making.

A notable strength of this study is the incorporation of CTG as a predictor in the ML-based model for LBW. Although CTG is routinely used for intrapartum fetal monitoring, it has rarely been included in predictive models for birth weight outcomes. Previous studies have suggested an association between LBW and abnormal CTG patterns [27,28]. In our analysis, abnormal CTG findings were significantly more common among LBW infants and emerged as one of the important predictors in the RF model. This suggests that fetal heart rate abnormalities may reflect underlying fetal compromise and placental dysfunction associated with impaired fetal growth and preterm birth. Integrating CTG with maternal and obstetric data extends our model beyond standard predictors, underscoring the clinical utility of routine intrapartum monitoring for better LBW risk stratification.

Limitations

This study has several strengths, including its prospective design, which enabled comprehensive data collection and minimized bias. However, it also has some limitations. First, the lack of external validation using datasets from different geographical regions or healthcare levels may limit the generalizability of our findings. Second, although multiple maternal and obstetric variables were included, some potentially important factors, such as maternal nutritional status, anemia during pregnancy, antenatal bleeding, genital tract infections, and placental pathology, were not available and may have influenced birth weight outcomes. Third, cardiotocography (CTG) findings were classified as normal or abnormal based on routine clinical interpretation, which may be subject to interobserver variability, and detailed quantitative CTG parameters were not analyzed. Finally, although SMOTE was applied only to the training dataset to address class imbalance, the model’s performance should be interpreted with caution, and prospective studies are needed to assess its clinical utility in real-world practice.

## Conclusions

In conclusion, the random forest model demonstrated superior predictive performance for LBW classification, achieving an accuracy of 87.1%, an AUC of 77.8%, and a high specificity of 90.9%. The most significant predictors identified by the model included preterm birth, primiparity (first-born status), absence of gestational diabetes, abnormal CTG finding, pre-eclampsia, and prior history of LBW. While these results highlight the model’s significant clinical applicability for early risk stratification and proactive neonatal care, its implementation in broader clinical practice should be preceded by external validation in diverse geographical and healthcare settings. Such validation is essential to ensure the model's reliability and generalizability across different populations before it can be fully adopted as a standard decision-support tool.
